# Sonic Hedgehog mimetic prevents leukocyte infiltration into the CNS during acute HIV infection

**DOI:** 10.1038/s41598-017-10241-0

**Published:** 2017-08-29

**Authors:** Vir B. Singh, Meera V. Singh, Dorota Piekna-Przybylska, Santhi Gorantla, Larisa Y. Poluektova, Sanjay B. Maggirwar

**Affiliations:** 1 Department of Microbiology and Immunology, University of Rochester Medical Center, 601 Elmwood Avenue, Box 672, Rochester, NY 14642 USA; 2 Department of Pharmacology and Experimental Neuroscience, University of Nebraska Medical Center, Omaha, NE 68198 USA

## Abstract

Infiltration of infected leukocytes culminates in establishment of a brain niche for Human Immunodeficiency Virus (HIV) during acute phase of infection, initiating an ongoing cascade of persistent viral replication and inflammation, that causes irreversible neuronal injury and HIV associated neurocognitive disease (HAND). In this study, humanized mice were treated with Smoothened Agonist (SAG), a Sonic Hedgehog (Shh) mimetic in order to fortify blood brain barrier (BBB) and dampen leukocyte extravasation into CNS during AHI. Results indicate that SAG treatment reduced viral burden in the CNS immediately after HIV transmission, but also conferred extended neuroprotection via increased BBB integrity (elevated levels of tight-junction protein, Claudin 5, and reduced S100B levels in periphery). These mice also showed healthier neurons with thick, uniform dendrites and reduced numbers of activated astrocytes. Additional *in vitro* experiments suggested SAG treatment was not associated with the establishment or reversal of latency in the target cells. Altogether, these findings validate neuroprotective role of Shh signaling and highlight the therapeutic potential of Shh mimetics against CNS complications associated with HIV infection. Further our results strongly demonstrate that pharmacological interventions to reduce leukocyte mobilization during early HIV infection, can provide prolonged neuroprotection, which might significantly delay the onset of HAND.

## Introduction

Human Immunodeficiency Virus (HIV) invades central nervous system (CNS) within the first few weeks of infection. This phase of infection, also known as acute HIV infection (AHI), is a 3 to 4-week period between HIV acquisition and seroconversion. It is marked by peak viremia, depletion of gastrointestinal CD4+ T cells, seeding of latent reservoirs and initiation and expansion of antiviral immune responses by the host^1^. A recent clinical study, which evaluated a small subset of high-risk Thai population (RV254/SEARCH 010) further confirmed that invasion of the CNS by HIV occurred as early as 8 days after estimated exposure^2^. In addition, neuro-invasion was associated with increased markers of inflammation and cellular infiltration, such as elevated cerebrospinal fluid (CSF) neopterin, and Choline/creatinine (tCHO/Cr) in the basal ganglia and occipital gray matter, at a median of 14 days after HIV transmission^2–5^. This early burst of inflammatory perturbation is a trigger which initiates the cascade of immune activation in the CNS. It also provides a basis for neurological injury and persistent infection in later stages of the disease, which manifests in HIV associated neurocognitive disease (HAND) in 30–50% of infected individuals despite anti-retroviral therapy (ART)^6, 7^.

While the virus enters CNS immediately, whether it is in the free virus form or via infected cells is a matter of debate. Multiple studies, support the “Trojan horse” model where virus enters through infected CD4+ T lymphocytes and/or monocytes during routine surveillance and infects CNS resident macrophages and microglia^8^. Studies in rhesus macaques treated with Natalizumab, which prevents leukocyte migration across endothelial barriers, showed profoundly reduced levels of HIV DNA in brain tissue as compared to untreated animals. These experiments did not utilize ART, hence CNS entry in the form of free virus particles was not affected, indicating that virus trafficking by immune cells, seems to be the preferred route of neuro-invasion^9, 10^. Further, changes in brain metabolism during the first year of HIV infection were found to correlate with increased numbers of CD16+ inflammatory monocytes indicating that monocyte dynamics in the periphery might affect CNS outcomes^3^.

Leukocyte transmigration into the CNS is predominantly regulated by the blood brain barrier (BBB), which comprises of specialized endothelial cells (ECs) that are interconnected by tight junctions as well as astrocyte end-feet and pericytes^11, 12^. Our group has recently shown that administration of Smoothened Agonist (SAG), a small molecule Sonic Hedgehog (Shh) mimetic, to chronic HIV-infected humanized mice resulted in increased BBB integrity and reduced neuro-inflammation, probably by preventing excessive infiltration of activated/infected leukocytes into the CNS^13^. Shh signaling is activated by binding of Shh to its receptor Patched (PTCH) that causes release of Smoothened (SMO), which then modulates further downstream targets such as tight-junction proteins and adhesion molecules via the transcription factor Gli-1^14^. SAG is a chlorobenzothiophene-containing agonist of SMO, that acts independent of PTCH and is as potent as processed N-terminal fragment of Shh^15^. It has previously been used to prevent Glucocorticoid induced Neonatal Cerebellar Injury^16^. We and others have also described a neuroprotective role for Shh signaling in BBB homeostasis^13, 17^, Our previous report using HIV infected humanized mice underscored the significant connection between Shh signaling and neuro-inflammation, neuronal injury and its subsequent culmination in HAND. Most importantly, these studies brought forward the notion that pharmacologic modulation of Shh signaling can be an interesting avenue of research towards the development of an adjunct therapy for HAND, which is currently unavailable^18–20^.

It also gave rise to another interesting notion that enhancing BBB integrity during AHI might reduce immune cell infiltration into the CNS, thereby attenuating the establishment of a CNS niche for HIV. Hence, in this report, humanized mice were used as a model to depict CNS related events happening within a few days of HIV infection, i.e. during AHI, marked by active viral replication in absence of adaptive immune responses. The mice were treated with a dose of SAG, before and after HIV infection and were sacrificed either on day 2 or on day 35 post infection. Mice treated with SAG showed significantly reduced leukocyte infiltration into the CNS on day 2, as well as reduced viral load in the brain on day 35. SAG treatment during AHI also resulted in better neuroprotection in terms of increased BBB integrity, reduced astrogliosis and morphologically healthier neurons, as a consequence of reduced leukocyte mobilization early on. Further we did not detect any discernible effect of SAG on establishment or reversal of latency in target cells. Our findings clearly highlight the therapeutic potential of Shh mimetics against CNS complications associated with primary HIV infection.

## Results and Discussion

### Activation of Shh signaling by SAG significantly reduces leukocyte migration into mouse CNS during acute phase of HIV infection

It is becoming increasingly clear that pathologic processes initiated during acute phase of HIV infection exert a strong influence on disease progression and comorbidities, such as HAND, associated with the chronic phase. The notion that therapeutic interventions during primary HIV infection can be an effective strategy to ameliorate the severity of disease in chronic stage has many precedents. Initiation of ART during early stages of HIV infection has proven beneficial in terms of improved brain metabolite levels and neuropsychological testing as well as preservation of mucosal Th17 function^5, 21–23^. A similar approach to early intervention was used in the current study to dampen the establishment of a CNS niche for HIV by strengthening the BBB with the use of an Shh mimetic, SAG.

**Figure 1:**
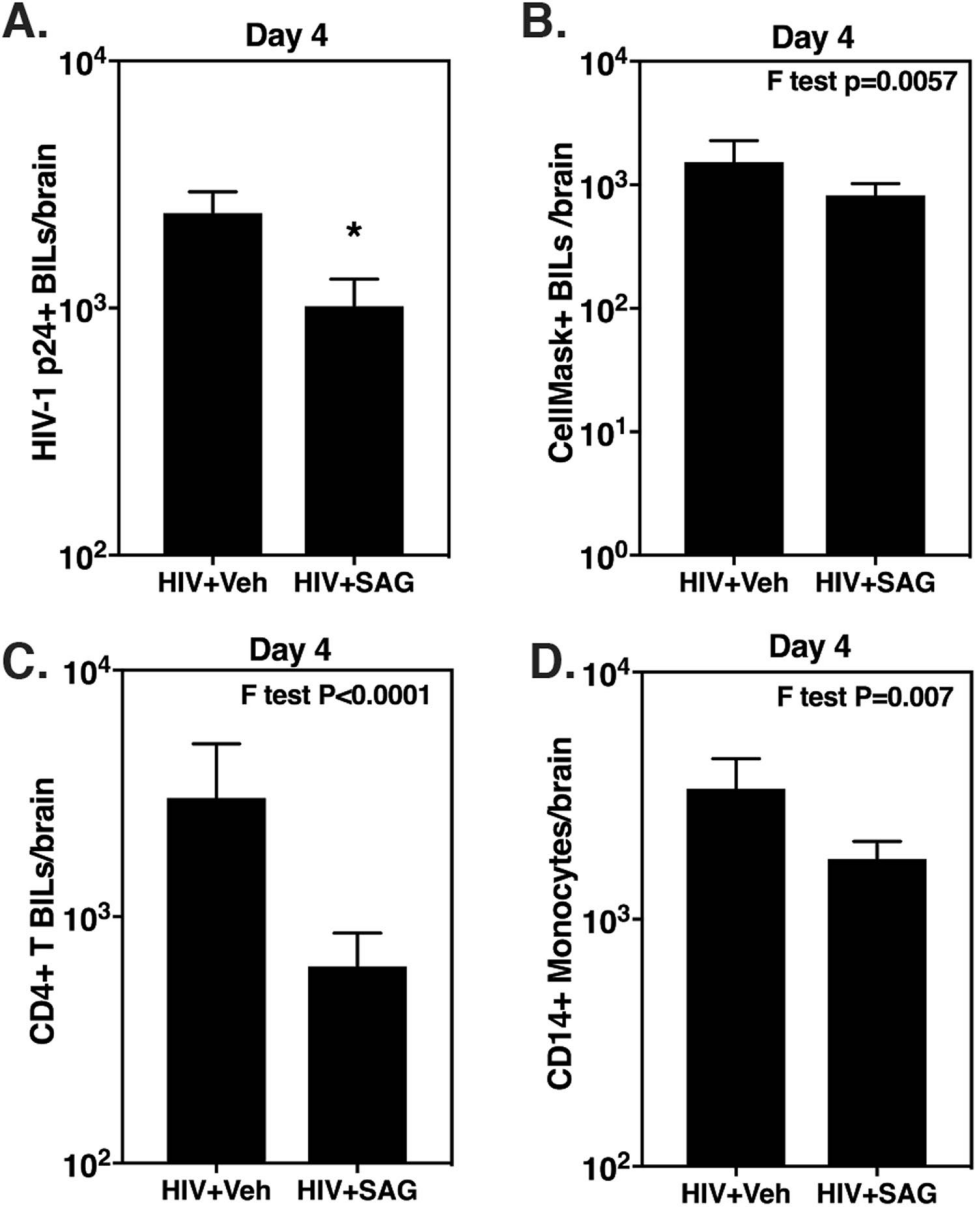
SAG mediated reduction in CNS leukocyte infiltration during acute HIV infection. Humanized CD34^+^-NSG mice were pretreated with SAG (20 μg/g body weight) (N = 5) or vehicle control (PEG 400 in PBS; N = 5) via i.p followed by injection with CellMask Red labeled HIV-infected PHA blasts (2 × 10^6^ cells/mouse) i.p. 24 hours later. Mice were given another dose of SAG on the next day and were sacrificed on day 4. Brain infiltrating leukocytes (BILs) were isolated and analyzed using flow cytometry to measure (**A**) p24+ BILs (**B**) CellMask Red+ BILs (**C**) CD4+ T cells and (**D**) CD14+ Monocytes. There was a significant decrease in p24+ BILs in SAG treated mice and similar trend was shown by all other cell types. Detection of CellMask Red+ cells in brain indicated that the founder cells were able to cross BBB. *Indicates p < 0.05 by unpaired t test.

A pioneer study by Alvarez *et al*. conclusively showed the involvement of Shh signaling in BBB homeostasis in wild-type mice^17^. In accordance, we have previously shown that Shh signaling is down-regulated in chronically infected humanized mice and its induction via SAG decreased neuro-inflammation^13^. However, the main caveat of this study was that, while SAG administration during chronic infection significantly reduced neuro-inflammation and subsequent neuronal injury, it failed to revert the neuronal damage already caused by almost 9 weeks of infection. With that in mind, the present study aimed to investigate if proactive BBB fortification via SAG can prevent/suppress the infiltration of infected/activated leukocytes into the brain during acute infection and prevent subsequent neuronal injury in later stages of the disease. For this, humanized mice were treated with a dose of SAG (20 μg/g body weight) before and after HIV infection at the interval of 18–20 hours between each administration. The mice were infected with CellMask Red (a membrane dye)-labelled HIV infected PHA blast cells in order to track if the founder cells extravasate into the CNS. On day 4 after the first SAG dose, the mice were sacrificed, perfused and brains were used to isolate infiltrating leukocytes. SAG treated animals had significantly less numbers of p24+CD4+ T cells in the brain (Fig. 1A, N = 5 in each group, p = 0.047). Interestingly we were able to detect CellMask Red+ cells in the brain at day 4, further confirming that HIV does invade the CNS within a few days of transmission (Fig. 1B)^2^. The ability of SAG to reduce leukocyte migration into the CNS was not specific to HIV infected cells, as there was a decrease in the total numbers of CD4+ T cells (Fig. 1C) and CD14+ monocytes (Fig. 1D) in brains of SAG treated mice. While the difference between CellMask+ BILs, total CD4+ T cells and monocytes among the two treatment groups was not statistically significant by t test, they showed a decreasing trend in the SAG treated group.

### Reduced leukocyte infiltration into CNS during primary HIV infection confers extended neuroprotection in SAG treated mice

**Figure 2:**
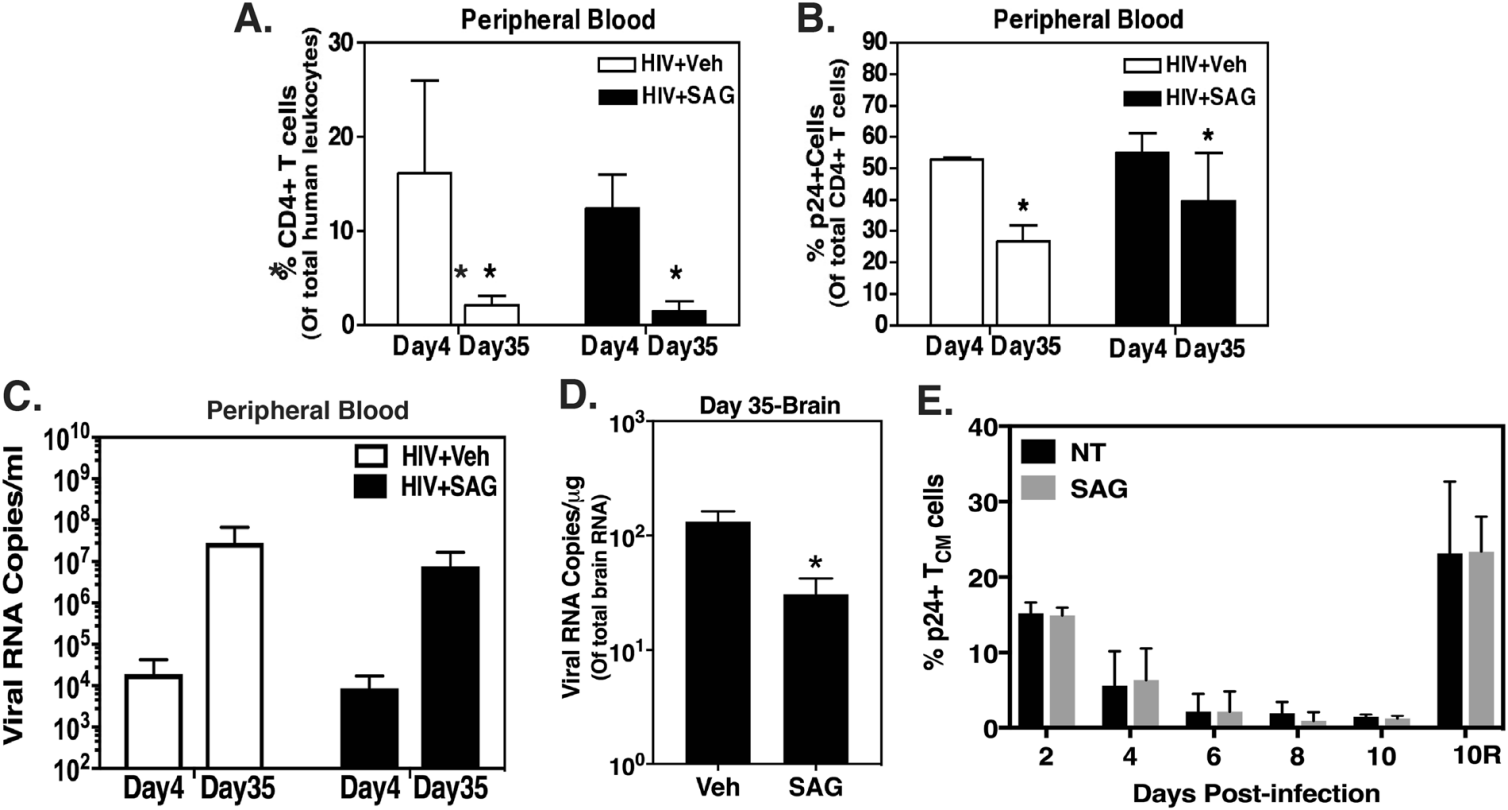
SAG treatment in primary HIV infection causes prolonged decrease in CNS viral burden. Whole blood obtained from HIV infected mice with or without SAG treatment was analyzed by flow cytometry to enumerate (**A**) total CD4+ T cells and (**B**) p24+ CD4+ T cells at Day 4 and Day 35 post-SAG pretreatment (N = 3 per group per time point). (**C**) Plasma samples and (**D**) total brain RNA from a few representative mice were analyzed by Cobas Ampliprep to measure viral RNA copies (N = 3–5 per group per time point). *Indicates p < 0.05 by unpaired t test (**E**) % p24+ cells in *in vitro* cultured T_CM_ cells infected with HIV.

In order to examine if decrease in migration of leukocytes into the brain during early HIV infection had any long-lasting effects on the BBB and CNS, parallel groups of mice were terminated on day 35 (i.e. almost 5 weeks, a midpoint to our previous chronic SAG study)^13^. Whole blood obtained from these mice was analyzed by flow cytometry to measure the percentage of circulating CD4+ T cells and p24+ CD4+ T cells. There was a significant decrease in CD4+ T cell percentages in mice comparing day 4 and 35 in mice treated with or without SAG (Fig. 2A, N = 3 per group, p = 0.003), indicating that the disease progression in periphery was not different among the groups. The decrease in p24 + CD4+ T cells on day 35 was probably reflective of the severe decrease in the total number on CD4+ T cells (Fig. 2B, N = 3 per group, p = 0.043). While the viral RNA copy levels in plasma showed an increasing trend in both the vehicle and SAG treated mice (N = 3–5 per group, p = 0.0534), there was a significant decrease in the viral RNA levels in SAG treated brains (Fig. 2C and D, N = 3 per group, p = 0.03). Further *in vitro* experiments were performed to rule out a possibility whether the observed reduction in CNS viral burden was due to diminished infiltration of infected leukocytes into the CNS early on during the infection and not because of any un-anticipated effect of SAG on establishment of viral latency. *In vitro* cultured T_CM_ (Central Memory CD4+ T) cells were infected with HIV and allowed to become latent in presence or absence of SAG. The cells were analyzed every alternate day (Day 2 being 48 hours post infection), for intracellular p24 expression and no effect of SAG was observed on establishment of latency (gradual decrease in intracellular p24 levels, Fig. 2E, N = 3 per group per time point). On Day 8, 1 million cells were reactivated with CD3/CD28 beads in present of SAG. As seen in data point 10 R of Fig. 2E no discernible effect of SAG was observed on reversal of latency in these cells. This evidence conclusively indicates that SAG-mediated reduction in CNS viral burden during later stages of the infection is indeed due to reduced migration of infected leukocytes in acute stage of the disease.

### Preemptive Shh activation via SAG is beneficial in maintaining BBB integrity as measured by tight junction protein expression and S100B levels despite progressive HIV infection

**Figure 3:**
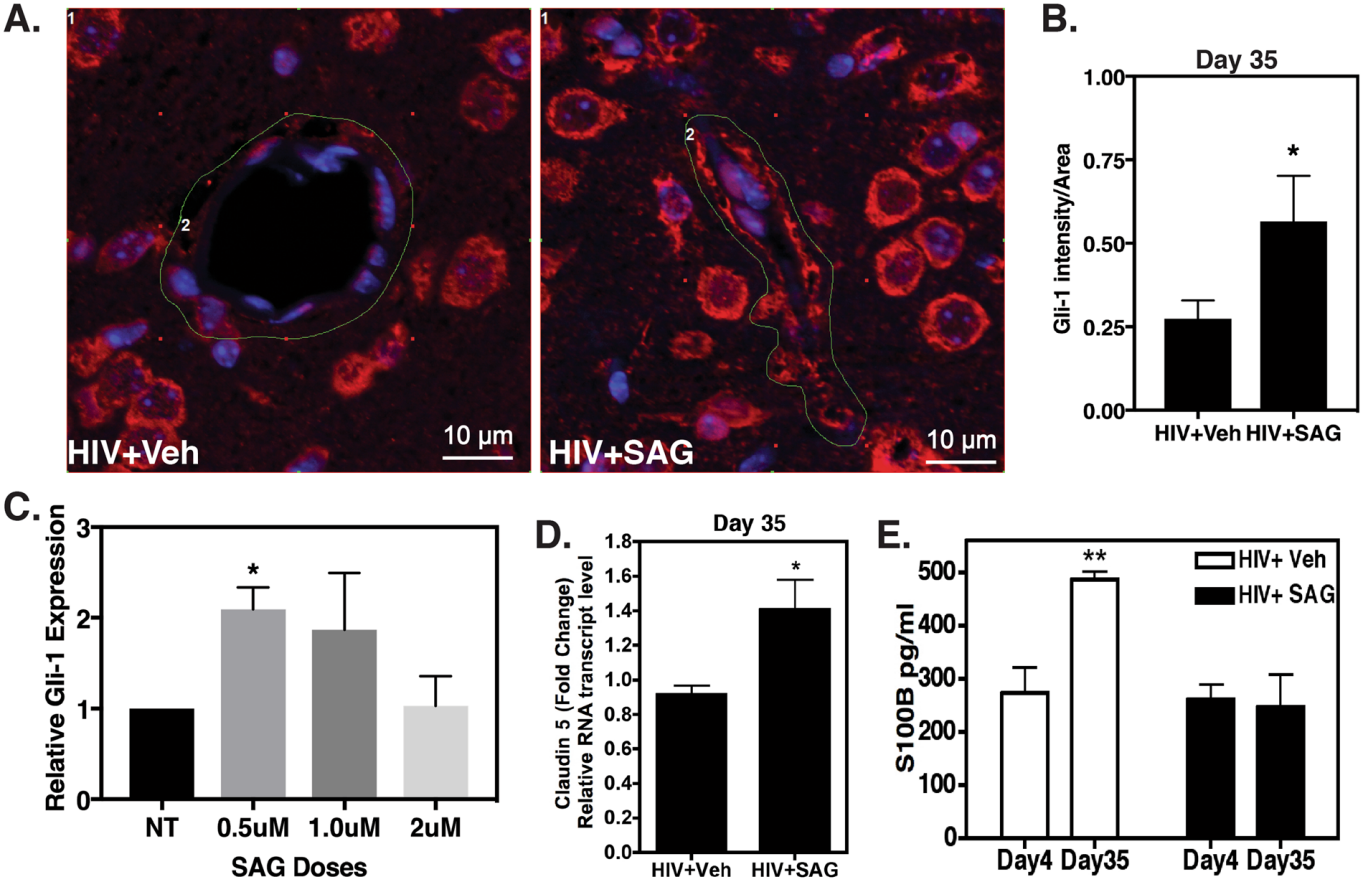
BBB fortification by SAG treatment during primary HIV infection is sustained in later stages of the disease. (**A**) Paraffin sections of representative brains obtained on day 35 post SAG pretreatment, were labeled with anti-Gli-1 (red) and Dapi (Blue). Images were taken from cortical region at 40X zoom 3. (**B**) Relative Gli-1 intensity in the outlined regions of interests (in green) from three representative images per group (**C**). Relative Gli-1 expression levels in SAG treated HBMECs by RT-QPCR (N = 3 per group) (**D**). Claudin 5 RNA levels by RT-QPCR (N = 3 per group) on day 35. (**E**) S100B levels in plasma as measured by ELISA (N = 3–5 per group per time point). *Indicates p < 0.05 and **Indicates p < 0.01 by unpaired t test or 1-way ANOVA and 2-way ANOVA respectively.

Astrocytes and neurons secrete Shh in adult brain, which then binds to its receptor Patched (PTCH) on microvasculature endothelial cells. Shh-PTCH interaction causes Smoothened (SMO) activation, which in turn leads to increased expression of tight junction proteins (e.g. Claudin 5), through Gli-1 transcription factor^24^. SAG bypasses Shh to directly activate SMO and the down-stream proteins Gli-1 and Claudin 5^14^. Therefore, we assessed the expression level of Gli-1 in mouse brains, 35 days post infection. There was a significant increase in Gli-1 expression in the endothelial cells in SAG treated brains (Fig. 3A and B, N = 2–3 per group, p = 0.028). In order to test if this effect of SAG on Gli-1 expression could be recapitulated in human cells, primary brain microvasculature endothelial cells (HBMECs) were treated with increasing doses of SAG (0.5–2 μM) for 24 hours and Gli-1 mRNA levels were measured by RT-QPCR. Maximum Gli-1 mRNA expression was observed in cells treated with 0.5 μM SAG, while cells treated with 2 μM SAG expressed Gli-1 levels comparable to non-treated cells (Fig. 3C, N = 3 per group, p = 0.0287). These results corroborate an earlier report by Chen *et al*.^15^. Next we measured RNA transcript levels of Claudin 5, a downstream target of Gli-1 in SAG treated brains and found that it was significantly elevated in treated brains (Fig. 3D, N = 3 per group, p = 0.042). These results indicate that SAG mediated induction of Shh signaling in AHI was sustained as the infection progressed and resulted in elevated levels of Tight junction protein expression. Next we determined if up-regulated Shh signaling was associated with a more functional, tighter BBB by measuring the levels of S100B, an astrocyte origin protein in peripheral plasma. Increased S100B levels in plasma are known to correlate positively with BBB leakage^25^. Indeed, while the S100B levels were increased significantly in vehicle treated mice from day 4 to day 35, they were unchanged in the SAG treated mice (Fig. 3E, N = 3–5 per group, p = 0.006), indicating that SAG treated BBB was impermeable to leukocytes.

### SAG treatment during AHI prevents astrogliosis and neuronal injury

**Figure 4:**
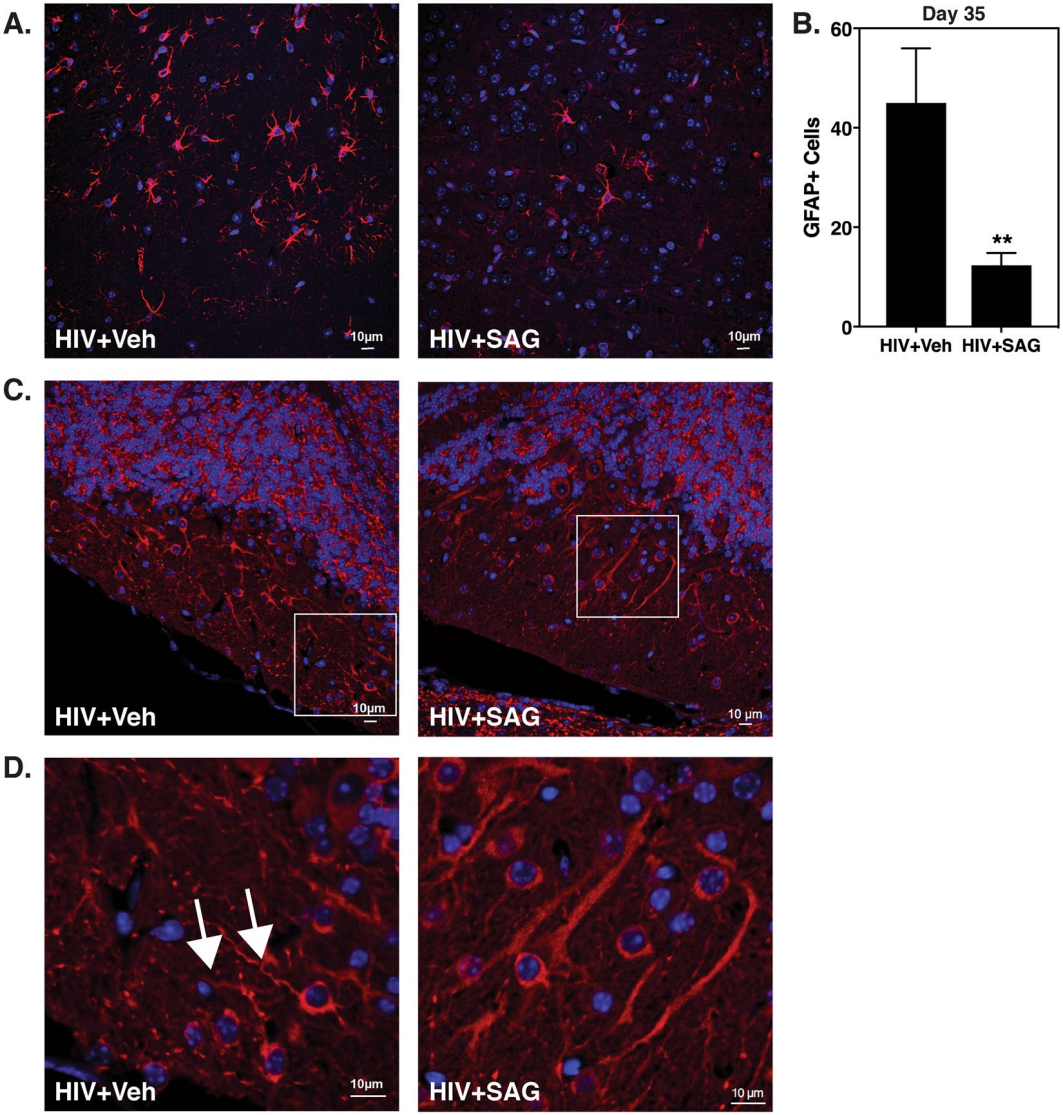
Increased Shh signaling in primary HIV infection results in decreased neuro-inflammation and neuronal damage Paraffin sections of representative brains obtained on day 35 post SAG pretreatment, were labeled with (**A**). anti- GFAP (red), an astrocyte activation marker and (**B**). GFAP+ cells were enumerated using Particle Analysis tool from ImageJ software from three random fields of view. (**C**) anti-MAP2 (red), a neuronal marker and Dapi (blue). Images were taken from cortical region at 40X and (**D**). regions enclosed in a white box in Fig. 4C were zoomed 3 times. White arrows indicate beaded, thin dendrites in brains without SAG treatment.

Next we sought to determine if the enhanced BBB integrity established a more neuroprotective environment in SAG treated brains. For this, we performed immune-histochemical analysis of brain cortex to evaluate astrocyte activation i.e. GFAP expression levels (a marker of neuro-inflammation) as well as neuronal morphology as seen by MAP2 expression. MAP2 is a microtubule assembly protein that plays a crucial role in determining and stabilizing dendritic shape in post-mitotic neurons^26^. As expected, SAG treatment lead to significantly reduced levels of GFAP expressing astrocytes (Fig. 4A and B). Although we do not see significant difference in the intensity of MAP2 expression (data not shown), SAG treated mouse brains showed thicker and uniformly shaped neuronal dendrites as compared to thin and beaded dendrites in vehicle treated mouse brains (Fig. 4C and D). Overall our findings show that Shh induction by SAG treatment reduced leukocyte infiltration into the CNS during AHI and also conferred extended neuroprotection for up to 35 days, with respect to CNS viral burden, BBB integrity, neuro-inflammation and neuronal injury.

This report adds significant new information complimentary to our previous report^13^ in which we showed neuroprotective effect of Shh signaling during chronic HIV infection. However, as stated before, SAG treatment during chronic infection failed to reverse the already incurred neuronal injury. We addressed this limitation by proactively inducing Shh activation via SAG before HIV infection in the present study. Secondly, in the earlier report, we were not able to provide direct evidence linking BBB tightening by SAG with reduction in the CNS infiltration by leukocytes, which was achieved here by measuring brain infiltrating leukocytes. Most importantly, since HIV-infected PHA blasts labeled with CellMask Red membrane dye were used as the mode of HIV infection, we were able to track the entry of founder cells injected intraperitoneally, into the CNS just within 48 hours of injection and substantiate that HIV indeed infiltrates the CNS very early during infection.

Further these findings are very significant because, as of now there are no (even partially successful) treatment options for HAND^18–20^. There is an ever increasing need for these therapeutics in order to improve the life-quality of HIV infected individuals, as they are living near-normal life spans with the advent of ART. Even the less severe forms of HAND, which are more prevalent in the post-ART era, have substantial effect on day-to day functions^27^. Treatments like SAG that delay the onset of neurocognitive impairment could potentially serve as functional cure for HAND. The second important conclusion from this study is the unequivocal merit to early-intervention especially with respect to neuronal complications. While ART buffers from the key pathologic indices i.e. sufficiently elevated CD4+ T cell numbers and undetectable viral loads, certain disease processes are irreversible such as the loss of mucosal CD4+ T cells^28^, neuronal injury^29^ and establishment of latent reservoirs^30^, especially in the CNS. It needs to be further investigated if combination treatment with ART and SAG protects from neuronal injury and prevents the establishment of the CNS niche. In addition, in-depth characterization of SAG, or similar Shh agonists like Purmorphamine, as therapeutic candidates for HIV associated complications of the CNS is also warranted.

## Materials and Methods

### Ethics statement

Mouse experiments were carried out in accordance with the Animal Welfare Act and the National Institute of Health (NIH) guidelines, and the University Committee on Animal Resources of the University of Rochester Medical Center approved the animal protocol (protocol # 2005-161). The Research Subjects Review Board at the University of Rochester Medical Center approved studies involving human samples. All the study participants were adults and blood samples were obtained after written informed consent, in accordance with the Declaration of Helsinki.

### Preparation of HIV-infected PHA blasts

PHA blasts were generated as per the ACTG Laboratory manual version 1.0 and were infected with HIV-1 BAL (Zeptomatrix Corp. NY) at the MOI (multiplicity of infection) of 1. A fraction of cells (0.1 × 10^6^) were fixed using 4% paraformaldehyde (PFA) and permeabilized using IC Perm (Invitrogen Inc, USA), followed by staining with antibody against p24 FITC (Beckman Coulter Inc. USA) and anti-human CD4 PE on a daily basis. The cells were analyzed using Accuri C6 flow cytometer to measure the percentage of p24 + CD4+ T cells. Once the p24 + CD4+ T cells reached to 60% of all viable cells, blasts were labelled with CellMask Red membrane dye (Invitrogen Inc. USA) as per the manufacturer’s instructions and used for infecting mice.

### SAG treatment and HIV infection of humanized mice

Humanized CD34^+^-NSG mice were generated as reported earlier^31^. 20–22 week old mice were administered SAG (20 μg/g body weight, Cayman Chemicals, USA) or vehicle control (PEG-400 in PBS, Sigma Aldrich, USA) intraperitoneally (i.p.). The mice were injected i.p. with HIV-infected PHA blasts (2 × 10^6^ cells/mouse) 18–20 hours later. 24 hours post-infection, the mice were given another dose of SAG and were sacrificed on the next day (day 4), or on day 35. Whole blood was collected by cardiac exsanguination. The mice were then perfused and brains were harvested. Blood was either used to isolate plasma or to perform flow cytometry to estimate cell counts for CD4+ T cells and p24 expression as described above (N = 3 per group). Plasma was used to perform S100B ELISA (as per manufacturer’s instructions, #SEA567Mu, Clone-Cloud Corp; N = 3–5 per group) or for viral RNA measurements (N = 3–5 per group). Levels of viral RNA copies/ml were analyzed with the automated COBAS Ampliprep system V2 (Roche Molecular Diagnostics, Mannheim, Germany) as described^31^. Brains were used for RNA extraction (N = 3 per group), immune-histochemical analyses (N = 2–3 per group) or isolation of brain infiltrating leukocytes (BILs; N = 5 per group).

### Isolation and quantitation of brain infiltrating leukocytes

BILs were isolated as previously described^32^ and resuspended in 100 µl PBS and fixed as described above. The cells were stained with antibodies against human CD4-PE and CD14 PE Cy 7 (a monocyte marker). The cells were then permeabilized using IC Perm and stained with anti-p24 FITC. The number of BILs were measured based on forward and side scatter and the expression of specific surface markers using flow cytometry (Accuri Cytometers, Ann Arbor, MI). CellMask Red+ cells are detected in channel 4 (FL4) and their percentage in BILs was enumerated to estimate the number of injected cells (i.e. founder cells) that had transmigrated into the CNS.

### *In vitro* culture of Central Memory CD4+ T cells (T_CM_) and HIV infection

T_CM_ cells were infected with single cycle VSV-G pseudotyped HIV-1 isolate and were cultured to establish latency as described previously^33, 34^. Briefly, naïve CD4+ T cells isolated from HIV uninfected healthy donors were cultured with a cytokine cocktail containing 10 ng/ml TGF-β, 2 μg/ml IL-12, 1μg/ml IL-4 and CD3/CD38 Dynabeads for three days. The cytokine cocktail and the dynabeads were then removed and replaced with 30U/ml of IL-2. After three more days, the cells were infected with HIV (0.5μg p24/million cells) by spinoculation. Cells were treated with 500ng/ml SAG one day before and 1 day after HIV infection (as done in the mouse experiments) or were left untreated. Cells were harvested 48 hours later (labelled as Day 2 in Fig. 2E) and fixed/permeabilized using BD Cytofix/Cytoperm reagent followed by staining with 5μl/million cells of anti-p24 FITC antibody. Stained cells were acquired on Accuri C6 Flow Cytometer. The staining was repeated every alternate day for 6 more days. By this time the cells acquire a latent phenotype (i.e. p24 negative). On Day 8, one million non-treated and SAG treated cells were reactivated to reverse latency using CD3/CD28 Dynabeads in presence of 500ng/ml SAG. The cells were analyzed for p24 expression on Day 10. All the cytokines were purchased from R&D systems.

### SAG treatment of Primary human brain microvasculature endothelial cells (HBMECs)

Cells were treated with treated with 0.5 μM, 1 μM and 2 μM SAG for 24 hours. Gli-1 RNA levels were measured by RT-QPCR as described previously^13^. The primers used for this experiment are from, Biorad prime PCR assay (Catalog # 10025636, Gli-1: qHsaCED0043346, GAPDH: qHsaCED0038674).

### Immunohistochemistry

Brains from representative mice in each group (N = 2–3 per group) were post-fixed overnight followed by paraffin embedding. 5 μm thick sections from cortex were immuno-stained with MAP2 (catalog# AB5622; Millipore, Darmstadt, Germany; 1:500), GFAP (catalog# ab7260; Abcam, 1:5000), and Gli1 (sc-20687; Santacruz, 1:100) and processed as described previously^13^. ImageJ software was used to quantify IHC data in Fig. 3 and 4 as described previously^13^.

### Statistical analysis

Graphpad Prism version 7 was used to perform all statistical analyses. Whole blood cell percentages, S100B levels and % p24+ T_CM_ cells were analyzed using 2-way ANOVA and Tukey post-test. Gli-1 RNA levels with different SAG doses was analyzed using 1-way ANOVA. All other comparisons were done using unpaired t test to compare group means or group variance respectively. Statistical significance by t test or 2-way ANOVA is indicated in the figures as *p < 0.05, **p < 0.01, and ***p < 0.001.
